# Detection and application of neurochemical profile by multiple regional ^1^H‐MRS in Parkinson's disease

**DOI:** 10.1002/brb3.792

**Published:** 2017-08-13

**Authors:** Jitian Guan, Yu Rong, Ye Wen, Huanze Wu, Hong Qin, Qingying Zhang, Wei Chen

**Affiliations:** ^1^ Department of Neurology the Second Affiliated Hospital of Shantou University Medical College Shantou Guangdong China; ^2^ Department of Preventive Medicine Shantou University Medical College Shantou Guangdong China

**Keywords:** N‐acetylaspartate (NAA), Parkinson's disease, proton magnetic resonance spectroscopy

## Abstract

**Introduction:**

The accurate diagnosis and monitoring of idiopathic Parkinson disease (PD), a progressive neurodegenerative disorder, has not been fully developed. This study sought to identify a neurochemical profile in multiple regions of the PD brain and healthy controls by proton magnetic resonance spectroscopy (^1^H‐MRS). We aimed to track changes of the brain neurochemical, quantify neuronal loss, and further determine the diagnostic value of ^1^H‐MRS.

**Methods:**

PD patients and healthy controls recruited from Second Affiliated Hospital of Shantou University Medical College, Shantou, southern China, underwent ^1^H‐MRS. Chemical information was obtained for ratios of N‐acetylaspartate to creatine (NAA/Cr), NAA to choline (NAA/Cho), and Cho to Cr for substantia nigra, globus pallidus, prefrontal lobe, hippocampus, cuneus gyrus, and dorsal thalamus regions.

**Results:**

Compared to the 20 healthy controls (12 male, age 58.75 ± 5.03 years), the 42 patients (21 male, age 61.60 ± 6.40 years) showed lower NAA/Cr and NAA/Cho ratios in substantia nigra, globus pallidus, prefrontal lobe, hippocampus, cuneus gyrus and dorsal thalamus regions (*p *<* *.01); NAA/Cr and NAA/Cho ratios were reduced for both patients with unilateral and mild/no cognitive impairment (*p *<* *.01); Unified Parkinson's Disease Rating Scale score was inversely correlated with NAA/Cr ratios in the substantia nigra (*r* = −.32; *p *=* *.042).

**Conclusion:**

NAA/Cr and NAA/Cho ratios may be useful metabolic biomarkers for early diagnosis of PD. Multi‐voxel ^1^H‐MRS can provide information on brain neurochemistry and may be a promising technique for diagnosis of and monitoring neuronal loss in PD.

## 1 INTRODUCTION

Parkinson disease (PD), characterized by progressive decline of the dopaminergic neurons inside the substantia nigra pars compacta, is the second most common neurodegenerative disorder. The disease features motor dysfunction and nonmotor symptoms including cognitive impairment and depression during the disease progression (Dauer & Przedborski, 2003; de Lau & Breteler, 2006). Currently, diagnosis is mainly based on clinical symptoms. However, motor symptoms are not unique to PD but also occur in other motor disorders such as cerebellar dysfunction (Sanes, Dimitrov, & Hallett, 1990). Additionally, the accuracy of clinical symptoms in diagnosis is less than 90% (Weingarten, Sundman, Hickey, & Chen, 2015).

The Unified Parkinson Disease Rating Scale (UPDRS) score (Goetz et al., 2008), the modified Hoehn‐Yahr scale, and the Montreal Cognitive Assessment (MoCA) scale (Gill, Freshman, Blender, & Ravina, 2008) are commonly used to assess the disease progression and cognitive impairment in PD patients. Nevertheless, a lack of objectivity may result in uncertainty in evaluation. Therefore, there is an urgent need for imaging techniques that can provide an alternative objective evaluating approach.

Proton magnetic resonance spectroscopy (^1^H‐MRS) can give a neurochemical profile of the brain for individuals with PD by quantifying a range of metabolites including N‐acetylaspartate (NAA), choline (Cho), and creatine (Cr) in different brain structures (Zanigni et al., 2015). Normally, the Cr concentration is stable and can be used as reference when assessing NAA and Cho concentration (Lucetti et al., 2001). Compared with healthy control subjects, PD patients showed reduced NAA/Cr ratio in the putamen (Watanabe et al., 2004), and temporal lobe (Levin et al., 2014) and increased Cho/Cr ratio in the posterior cingulate (Nie et al., 2013), so these two variables could be used as metabolic markers to reflect the neuronal function in the PD brain. Furthermore, compared with the putamen and cerebral white matter, the changes of NAA/Cr ratio are proven to be more pronounced in the pontine base in PD patients than controls (Watanabe et al., 2004). However, contradictory results for the NAA/Cr ratio in the frontal cortex have been reported in parkinsonian syndromes subjects (Abe et al., 2000). In addition, some studies showed no significant difference in Cho/Cr ratio in the lentifom nucleus between PD patients and healthy controls (Federico et al., 1997), which may result from regional and individual variability or different signal‐to‐noise ratio in different brain structures. Consequently, an overall description of the metabolic status in multiple regions of the brain is needed.

Previous studies have shown that the neurochemical profile produced with ^1^H‐MRS may provide useful information for the diagnosis, monitoring, and evaluation of treatment efficacy in PD. A randomized permuted block study (Mazuel et al., 2015) comparing patients with PD in drug‐on and drug‐off conditions and healthy controls found that total NAA and Cr were significantly lower in drug‐off patients than controls and further, the two biomarkers increased after dopaminergic therapy. In addition, MRS studies showed that PD involved widespread neuronal and axonal damage. Moreover, Wu et al. (2016) suggested a more notable change in NAA/Cr on the side affected at the onset of PD, which may be a reasonable interpretation to the asymmetric symptoms and signs in PD patients.

Given the background, we used a multi‐voxel ^1^HMR spectroscopic approach to provide a neurochemical profile of multiple regions of the idiopathic PD brain. We also recorded the UPDRS score, modified Hoehn‐Yahr stage, and the MoCA score of PD patients. This study aimed to assess the relationship between the NAA/Cr, NAA/Cho and Cho/Cr ratio and UPDRS score, Hoehn‐Yahr stage and MoCA score in PD patients to assess the value of ^1^H‐MRS in early diagnosis of the disease and evaluation of cognitive ability of PD patients.

## 2 MATERIALS AND METHODS

### 2.1 Subjects

This protocol was approved by the Ethics Committee of the Second Affiliated Hospital of Shantou University Medical College and was performed according to the Declaration of Helsinki. Written informed consent was obtained from all participants.

We included 42 PD patients (21 male, age 61.60 ± 6.40 years) admitted to the Second Affiliated Hospital of Shantou University Medical College for treatment between January 2012 and March 2015 and 20 healthy control subjects without neurologic disorders (12 male, age 58.75 ± 5.03 years) who were visiting the Center of Health Examination during the same time. All patients complied with diagnostic criteria and received a diagnosis from a specialized physician. We excluded patients and controls with (1) history of brain disease (cerebral infarction or cerebral hemorrhage), (2) presence of mental illness or metabolic syndrome, and (3) inability to provide consent.

Patients were assessed by the UPDRS score, modified Hoehn‐Yahr stage and MoCA scale. The Hoehn‐Yahr stage included stages 1 to 5, 1.5 and 2.5, with patients divided into unilateral impairment (Hoehn‐Yahr stage < 2) and bilateral impairment (Hoehn‐Yahr stage ≥ 2) groups, the MoCA score ranges from 0 to 30 points, and we subdivided the patients into cognitive impairment (<20 points) and mild/no cognitive impairment (≥20 points) groups. All participants underwent MRI and multi‐voxel ^1^H‐MRS examination.

### 2.2 MR imaging and ^1^H‐MRS

Routine MRI and ^1^H‐MRS were performed with a 3‐T MRI system (Sigma HDx Twin speed, General Electric Medical Systems) with a standard 8‐channel head coil. Two associate chief physicians interpreted all images.

The MRI parameters were: repetition time (TR), 4,420.0 ms; echo time (TE), 112.1 ms; matrix, 512 × 512; slice thickness, 5.0 mm; interlayer thickness, 1.0 mm.

Multi‐voxel ^1^H‐MRS were performed using a point‐resolved spectroscopy sequence with the following parameters: TR, 1,500 ms; TE, 35 ms; phase × frequency = 18 × 18, volume of interest, 8 × 10 × 1 cm; field of view, 240 × 240 mm; and number of excitations = 1.

**Figure 1:**
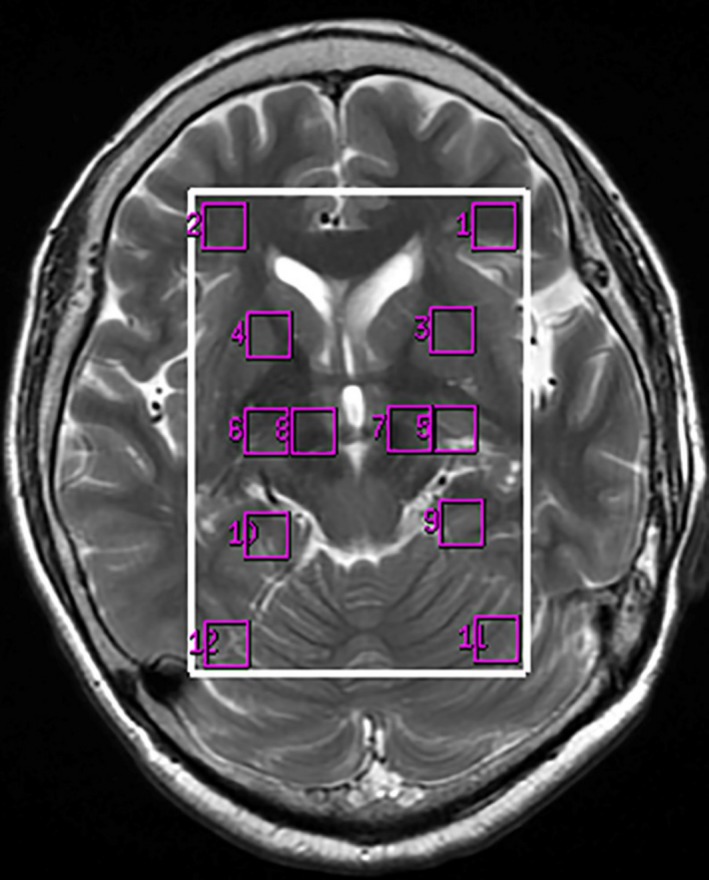
The regions of interest location on ^1^H‐MRS images (1, 2), dorsal thalamus (3, 4), globus pallidus (5, 6), substantia nigra (7, 8), hippocampus (9, 10), cuneus gyrus (11, 12)

Water suppression (>95%) and shimming (linewidth, <12 Hz) were automatically achieved using a variable pulse power and optimized relaxation delay scheme. After zero‐filling and baseline correction, the spectra were post‐processed automatically using “Advantage Window 4.6 workstation Functool software” (General Electric Medical Systems Signa excite 3.0T HD Ecospeed MRI, ADW 4.6 workstation, USA). The peak areas were the proportion using creatine as the reference which represent the various metabolic concentration such as NAA/Cr and Cho/Cr. The regions of interest (ROIs) for spectral analysis included the substantia nigra, globus pallidus, prefrontal lobe, hippocampus, cuneus gyrus and dorsal thalamus on both sides of the brain (Figure 1).

### 2.3 MRS analysis

**Figure 2:**
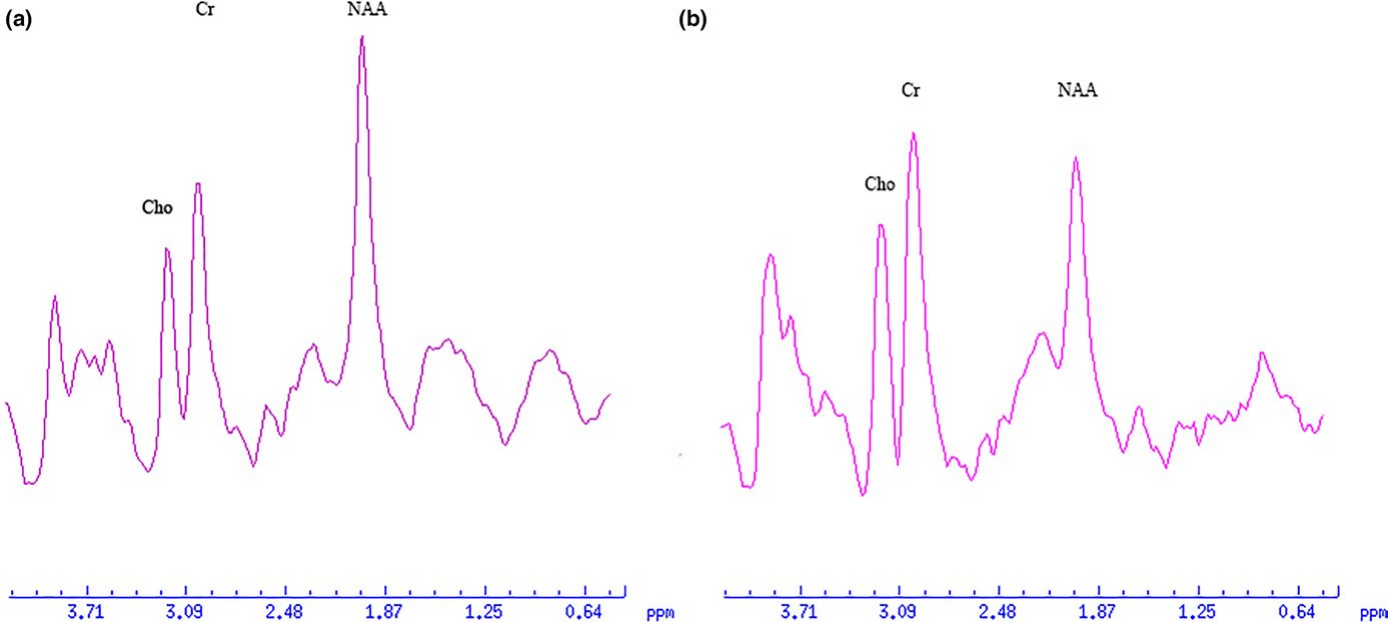
^1^H‐MRS spectra obtained from a control subject (a) and a PD patient (b)

**Figure 3:**
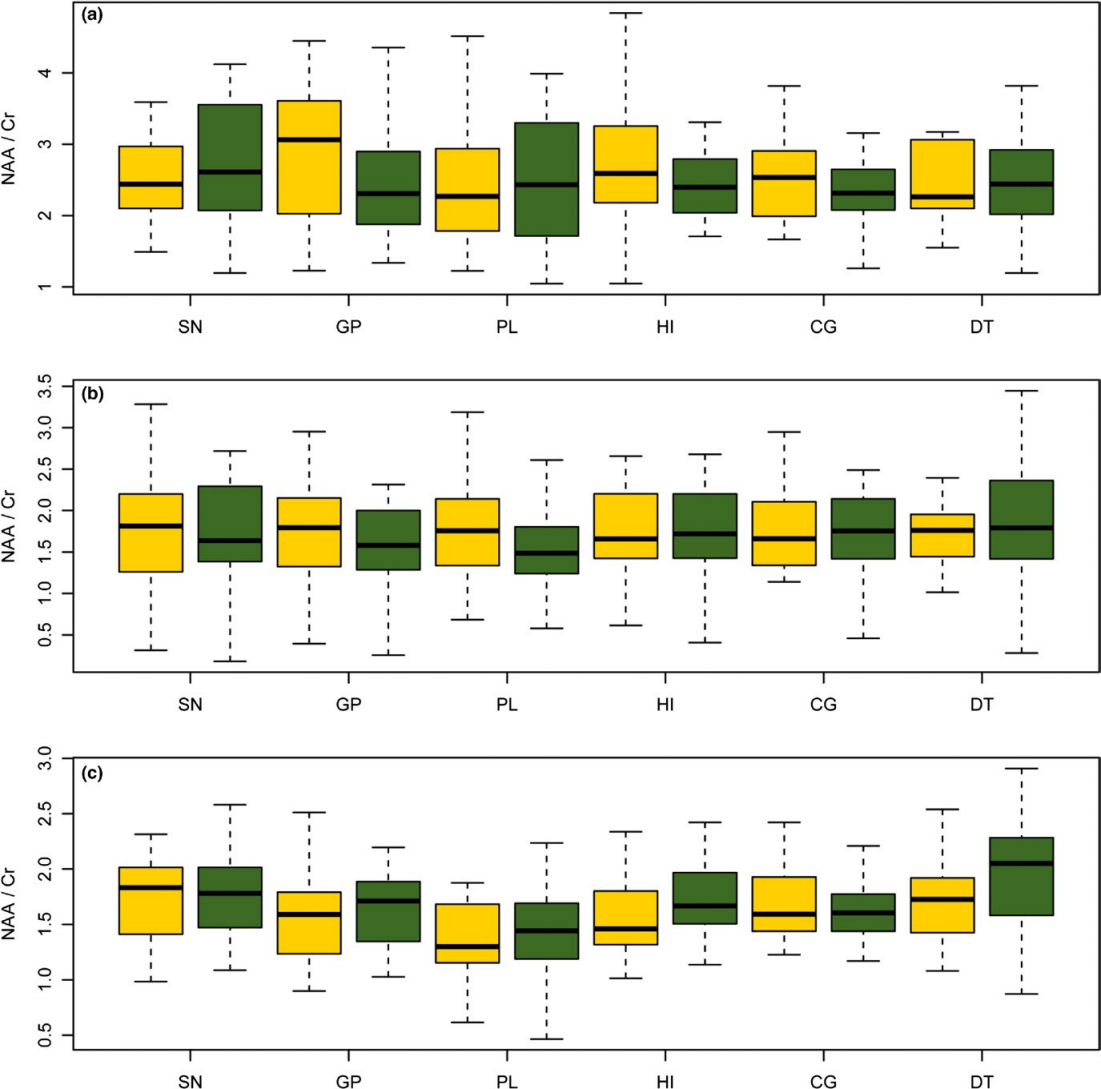
NAA/Cr ratios in substantia nigra, globus pallidus, prefrontal lobe, hippocampus, cuneus gyrus and dorsal thalamus for (a): healthy volunteers in left and right brain. (b): PD patients with unilateral impairment in the brain contralateral or ipsilateral to clinical sign and (c): PD patients with bilateral impairment in left and right brain

All spectra were transferred to an Advantage Window 4.6 workstation (GE, ADW 4.6 workstation, USA) and were processed using Functool (General Electric Medical Systems, USA). The metabolite signal intensity for NAA, Cho, and Cr were 2.02, 3.22 and 3.02 ppm, respectively. Examples of spectra are presented in Figure 2. Because of no significant differences between the left and right hemisphere for control and bilateral impaired PD patients and between the side contralateral and ipsilateral to for unilateral impaired PD patients, the values obtained for each side were averaged to represent the metabolism level for each participant (for NAA/Cr ratio, see Figure 3; for NAA/Cho and Cho/Cr ratios, see Figure S1 and Figure S2 online, respectively).

### 2.4 Statistical analysis

Quantitative data are presented as mean (SD), and categorical data as number (%). Comparisons for metabolism level between cases and controls were assessed by Mann–Whitney test. Kruskal–Wallis H followed by Dunn–Bonferroni post hoc method were used for comparisons for three groups and pairwise comparison between three groups.

Relations between metabolism level and duration of disease, UPDRS score, Hoehn‐Yahr stage, and MoCA score in PD patients were studied by Spearman correlation analysis.

Statistical analysis involved use of SPSS 20.0 (SPSS, Inc, Chicago, IL, USA). Two‐sided, *p* < .05 was considered statistically significant.

## 3 RESULTS

Overall, 42 patients (61.60 ± 6.40 years) and 20 controls (58.75 ± 5.03 years) were enrolled in the study. For the cases, twenty (47.6%) had unilateral impairment and 22 (52.4%) bilateral impairment by Hoehn‐Yahr stage. Six patients (14.3%) had cognitive impairment and 32 (85.7%) mild/no cognitive impairment by MoCA score. Unilateral and bilateral impaired patients did not differ in sex or age, history of PD or MoCA score (all *p* > .05), but mean (SD) UPDRS score was higher for bilateral than unilateral impaired patients (22.85 ± 7.18 vs. 29.59 ± 9.29, respectively; *p* = .011). The baseline characteristics of the cases are in Table 1.

**Table 1:** Demographics and clinical characteristics of the Parkinson disease (PD) patients

Characteristics Mean (SD)	Cases (*n* = 42)	Unilateral (*n* = 20)	Bilateral (*n* = 22)	*p*‐value
Male (*n*, %)	21 (50.0)	10 (50.0)	11 (50.0)	1.000
Age (year)	61.60 (6.40)	60.25 (5.89)	62.82 (6.73)	.111
Disease duration (month)^a^	12 (6–36)	12 (6–12)	13.5 (6–36)	.252
Hoehn and Yahr stage	1.82 (0.62)	1.30 (0.25)	2.30 (0.45)	**<.001**
UPDRS score (range 0–147)	26.38 (8.93)	22.85 (7.18)	29.59 (9.29)	**.011**
MoCA score (range 0–30)	16.12 (5.07)	17.65 (5.54)	14.73 (4.27)	.150

P, comparing unilateral and bilateral PD by the corresponding statistical test; UPDRS, Unified Parkinson Disease Rating Scale; MoCA, Montreal Cognitive Assessment scale. The bold indicates a statistically significant association.

a Data are expressed in median (interquartile range).

The NAA/Cr, NAA/Cho, and Cho/Cr ratios for cases and controls in multiple regions are summarized in Table 2. Overall, NAA/Cr and NAA/Cho ratios were significantly lower in the substantia nigra, globus pallidus, prefrontal lobe, hippocampus, cuneus gyrus, and dorsal thalamus regions for total cases than healthy controls and lower in the six regions for both unilateral and bilateral impaired patients than controls, with no difference between unilateral and bilateral impaired PD patients (all *p* > .05).

**Table 2:** Metabolic status in multiple regions of the brain for control subjects and PD patients by impairment status

Region	Controls	Cases	Unilateral impairment	Bilateral impairment	Mild/no cognitive impairment	Cognitive impairment
NAA/Cr						
SN	2.57 (2.27–3.26)	1.80 (1.40–2.10)***	1.75 (1.41–2.28)***	1.84 (1.40–2.01)***	1.40 (1.27–2.19)**	1.85 (1.43–2.14)***
GP	2.84 (1.90–3.48)	1.65 (1.44–1.93)***	1.69 (1.43–2.12)***	1.60 (1.41–1.85)***	1.66 (1.23–2.21)*	1.65 (1.45–1.91)***
PL	2.43 (1.86–3.32)	1.52 (1.23–1.89)***	1.64 (1.30–2.06)***	1.44 (1.20–1.69)***	1.56 (1.27–2.18)	1.52 (1.21–1.86)***
HI	2.61 (2.16–3.22)	1.68 (1.48–1.92)***	1.77 (1.52–2.13)***	1.64 (1.47–1.87)***	1.77 (1.43–2.21)*	1.66 (1.48–1.91)***
CG	2.52 (1.95–2.76)	1.69 (1.53–1.88)***	1.80 (1.39–2.07)***	1.67 (1.53–1.81)***	1.83 (1.68–2.37)	1.68 (1.51–1.85)***
DT	2.56 (2.07–3.47)	1.85 (1.59–2.07)***	1.80 (1.54–1.99)***	1.88 (1.70–2.10)***	1.74 (1.45–2.17)*	1.87 (1.61–2.09)***
NAA/Cho						
SN	3.72 (2.81–4.83)	2.02 (1.74–2.74)***	1.98 (1.69–2.72)**	2.52 (1.77–2.86)**	2.01 (1.83–2.59)	2.02 (1.69–2.82)***
GP	3.65 (2.74–4.41)	1.89 (1.49–2.45)***	1.87 (1.36–2.40)**	1.95 (1.51–2.60)***	1.73 (1.32–2.67)*	1.91 (1.42–2.53)***
PL	3.43 (2.76–4.11)	1.76 (1.41–2.28)***	1.67 (1.46–2.39)***	1.80 (1.24–2.34)***	2.31 (1.79–2.89)	1.67 (1.28–2.14)***
HI	3.67 (2.86–4.02)	2.26 (1.73–2.66)***	2.07 (1.52–2.73)***	2.40 (1.94–2.64)***	2.52 (1.65–3.00)	2.20 (1.75–2.65)***
CG	4.08 (3.47–4.79)	2.27 (1.93–3.12)***	2.06 (1.76–2.75)***	2.33 (1.98–3.19)***	2.29 (1.89–3.39)*	2.27 (1.92–3.06)***
DT	4.01 (3.18–4.33)	1.96 (1.47–2.44)***	1.84 (1.46–2.21)***	2.20 (1.49–2.49)***	1.63 (1.30–2.37)**	2.12 (1.51–2.45)***
Cho/Cr						
SN	0.75 (0.55–0.88)	0.83 (0.66–0.97)	0.87 (0.67–0.97)	0.81 (0.65–0.98)	0.77 (0.67–0.92)	0.83 (0.64–0.98)
GP	0.83 (0.65–0.98)	0.92 (0.66–1.17)	1.01 (0.64–1.28)	0.89 (0.68–1.11)	0.96 (0.66–1.40)	0.92 (0.65–1.15)
PL	0.72 (0.58–1.00)	0.90 (0.65–1.17)	1.05 (0.71–1.33)	0.85 (0.61–1.10)	0.70 (0.62–1.05)	0.91 (0.68–1.20)
HI	0.72 (0.65–0.89)	0.75 (0.65–0.99)	0.90 (0.69–1.07)	0.70 (0.62–0.79)	0.70 (0.60–0.86)	0.75 (0.66–1.00)
CG	0.61 (0.51–0.77)	0.76 (0.56–1.01)*	0.88 (0.62–1.06)	0.73 (0.53–0.85)	0.88 (0.59–1.02)	0.74 (0.55–1.02)
DT	0.76 (0.57–0.94)	0.99 (0.78–1.32)**	1.04 (0.87–1.36)*	0.93 (0.76–1.30)**	1.09 (0.99–1.35)*	0.97 (0.75–1.34)*

NAA, N‐acetylaspartate; Cho, choline; Cre, creatine; SN, substantia nigra; GP, globus pallidus; PL, prefrontal lobe; HI, hippocampus; CG: cuneus gyrus; DT, dorsal thalamus.

The data are presented as median (interquartile range).*p < .05, **p < .01, ***p < .001, compared with controls.

Compared to controls, patients with mild or no cognitive impairment showed significantly reduced NAA/Cr ratios in substantia nigra, globus pallidus, hippocampus, and dorsal thalamus regions (Figure S1), whereas the NAA/Cho were lower in globus pallidus, cuneus gyrus, and dorsal thalamus regions. Patients with cognitive impairment showed significantly reduced NAA/Cr and NAA/Cho ratios in all six regions of interest, with no difference between cognitive and mild or no cognitive impairment patients (all *p* > .05).

Cho/Cr ratios in the cuneus gyrus were higher for all PD patients than control subjects. The differences were even greater in the dorsal thalamus and remained significantly different on stratification by Hoehn‐Yahr stage or MoCA score.

We observed modest but significant relationship between UPDRS score and NAA/Cho ratio in the substantia nigra (*r* = −.42; *p *=* *.006), globus pallidus (*r* = −0.37; *p *=* *.017), hippocampus (*r* = −.36; *p *=* *.021), and cuneus gyrus (*r* = −.38; *p *=* *.013). In addition, UPDRS score and NAA/Cr ratio were negatively correlated in the substantia nigra (*r* = −.32; *p *=* *.042). (For NAA/Cr ratios, see Figure S3, for NAA/Cho, see Figure S4.)

## 4 DISCUSSION

In thisstudy, we used a multi‐voxel ^1^H‐MR spectroscopic approach to describe a neurochemical profile in multiple regions of the PD brain. NAA/Cr and NAA/Cho ratios were lower in the substantia nigra, globus pallidus, prefrontal lobe, hippocampus, cuneus gyrus and dorsal thalamus regions of the brain of PD patients than healthy controls, which indicates widespread neuronal and axonal damage in the PD brain. Besides, NAA/Cr and NAA/Cho ratios were lower in PD patients with unilateral or mild/no cognitive impairment than controls, which suggests that those two metabolites might be early diagnostic biomarkers of PD and sensitive biomarkers of cognitive impairment in PD. In addition, NAA/Cr ratios in the prefrontal lobe and cuneus gyrus and NAA/Cho ratios in the substantia nigra, prefrontal lobe and hippocampus might indicate severity of cognitive impairment. We found high Cho/Cr ratios in the dorsal thalamus of PD patients. Moreover, the significant inversely correlation between UPDRS score and NAA/Cho ratios in the substantia nigra, globus pallidus, hippocampus, and cuneus gyrus and NAA/Cr ratios in the substantia nigra implying that those two metabolites from corresponding brain regions might be used to monitor pathologic changes in patients with PD.

Our ^1^H‐MRS study revealed significantly reduced NAA/Cr and NAA/Cho ratios in PD patients in multiple regions of the brain. Rodent PD models have shown that NAA is absent in mature glial cells and is a neuron‐specific molecule (Moffett, Namboodiri, Cangro, & Neale, 1991). Choe et al. (1998) demonstrated reduced NAA/Cr in PD patients and Holshouser et al. (1995) demonstrated significantly reduced NAA/Cho ratio in PD patients, and researchers have demonstrated that the level of NAA may reflect the status of neuron function. Specifically, reduced NAA level may represent reduced number of neurons or neuron deficiency, whereas a decreased NAA level reflects severe neurodegeneration and motor impairment (Sharma et al., 2013). Our results agree with previous researches, and the biomarker changes in PD patients in our study could point to neuronal loss or degeneration or neural mitochondrial dysfunction in the PD brain.

Although some studies found differences between the side ipsilateral and contralateral to clinical signs in PD patients with unilateral impairment, our study did not find significant differences between the contralateral and ipsilateral sides in multiple regions of the PD brain, and no significant differences between the left and right hemispheres in controls or bilateral impaired patients. The reason could be explained by the prior existence of pathologic changes to clinical symptoms. In addition to that, our results agree with previous studies (Mazuel et al., 2015). Besides, reduced NAA/Cr, and NAA/Cho ratios and increased Cho/Cr ratios in the side ipsilateral to clinical signs in patients with PD may clinically indicate the gradual development of bilateral symptoms in patients with unilateral impairment.

We observed widespread reduced NAA/Cr and NAA/Cho ratios in PD brain regions, including the substantia nigra, globus pallidus, prefrontal lobe, hippocampus, cuneus gyrus and dorsal thalamus. Our results fall in line with earlier neuroimaging ^1^H‐MRS studies, showing reduced NAA/Cr ratio in the temporoparietal cortex (Hu et al., 1999) and motor cortex (Lucetti et al., 2001). However, there are single‐volume ^1^H‐MRS gave conflicting results, which failed to demonstrate significant NAA/Cr ratio change in the striatum (Holshouser et al., 1995) and significant NAA/Cho ratio change in the occipital cortices (Tedeschi et al., 1997). The discrepancy can be explained in part by the difference in study population and sample size and the region of interest in previous studies. Further studies are needed to address this issue.

On the analysis stratified PD patients to unilateral and bilateral PD patients, there are differences in NAA/Cr and NAA/Cho ratios between PD patients with unilateral and bilateral impairment, and controls but not between unilateral and bilateral impairment patients. The fact that the Hoehn‐Yahr stage of our bilateral PD patients group ranged from 2 to 3 and treatment of PD might explain this absence of significant difference. The MoCA scale is a sensitive and brief test that can be used to screen mild cognitive impairment (Dalrymple‐Alford et al., 2010). When stratified PD patients into cognitive impairment and mild/no cognitive impairment, our results showed that NAA/Cr and NAA/Cho ratios were lower for our patients with mild/no cognitive impairment than healthy control subjects. Since the NAA/Cr ratios in the prefrontal lobe and cuneus gyrus and the NAA/Cho ratios in the substantia nigra, prefrontal lobe and hippocampus were statistically significant reduced in cognitive impairment but not mild/no cognitive impairment than controls, those information might be used to detect the severity of cognitive impairment. Additionally, previous observations showed that clinical symptoms will not appear until patients show >60% dopaminergic neuron loss, our results highlight the change in NAA/Cr and NAA/Cho ratios for patients with mild/no cognitive impairment and provide evidence of prior existence of the neurochemical changes to cognitive function in PD.

NAA/Cho ratios in the substantia nigra, globus pallidus, hippocampus, and cuneus gyrus and NAA/Cr ratios in substantia nigra were inversely correlated with the UPDRS score. The relatively weak correlation may due to the relatively small variations in UPDRS scores in our study population. However, our results are in keep with previous investigations (Wu et al., 2016) and the significant inverse relationship between the NAA/Cho and NAA/Cr ratios and the UPDRS score suggests that the reduction NAA/Cho and NAA/Cr ratios might be associated with advanced PD and thus, more pronounced neuronal damage.

Consistent with previous observations (Clarke & Lowry, 2000; Nie et al., 2013), the current ^1^H‐MRS study observe increased Cho/Cr ratios in the cuneus gyrus and dorsal thalamus of PD patients as compared with controls. However, studies have shown conflicting results: some studies found no changes in the frontal lobe (Abe et al., 2000; Watanabe et al., 2004) and others reported decreased Cho/Cr ratios in the temporoparietal cortex in PD patients (Taylorrobinson et al., 1999). Technical factors and different regions of interest could be possible explanations, and further ^1^H‐MR spectroscopic study is needed.

## 5 SUMMARY

PD has become a major public health problem worldwide and mostly affects the aging population. ^1^H‐MRS can be used to track changes in brain neurochemistry with the advantage of noninvasive. Since the aging of the population in China, the burden of disability due to PD is likely to increase in the near future, and it is important to have an improvement in the accuracy in PD diagnosis. In our study, we identified a neurochemical profile in multiple regions of the PD brain using multi‐voxel ^1^H‐MRS. Reduced NAA/Cr, and NAA/Cho ratios that were observed in PD patients in the substantia nigra, globus pallidus, prefrontal lobe, hippocampus, cuneus gyrus and dorsal thalamus showed widespread neuronal and axonal damage in the PD brain. Besides, the changes of NAA/Cr and NAA/Cho ratios in patients with mild/no cognitive impairment may provide the evidence that neurochemical changes are prior to the change in cognitive function in PD patient. In addition, the significant negative correlation between UPDRS score and NAA/Cr and NAA/Cho ratios may reflect the value of ^1^H‐MR spectroscopic in monitoring pathologic changes in PD.

## CONFLICTS OF INTEREST

None.

## References

[brb3792-bib-0001] Abe, K. , Terakawa, H. , Takanashi, M. , Watanabe, Y. , Tanaka, H. , Fujita, N. , … Yanagihara, T . (2000). Proton magnetic resonance spectroscopy of patients with parkinsonism. Brain Research Bulletin, 52, 589–595. 10974501 10.1016/s0361-9230(00)00321-x

[brb3792-bib-0002] Choe, B.‐Y. , Park, J.‐W. , Lee, K.‐S. , Son, B.‐C. , Kim, M.‐C. , Kim, B.‐S. , … Shinn, K. S . (1998). Neuronal laterality in Parkinson's disease with unilateral symptom by in vivo 1H magnetic resonance spectroscopy. Investigative Radiology, 33, 450–455. 9704284 10.1097/00004424-199808000-00005

[brb3792-bib-0003] Clarke, C. E. , & Lowry, M. (2000). Basal ganglia metabolite concentrations in idiopathic Parkinson's disease and multiple system atrophy measured by proton magnetic resonance spectroscopy. European Journal of Neurology: The Official Journal of the European Federation of Neurological Societies, 7, 661. 10.1046/j.1468-1331.2000.00111.x 11136352

[brb3792-bib-0004] Dalrymple‐Alford, J. C. , MacAskill, M. R. , Nakas, C. T. , Livingston, L. , Graham, C. , Crucian, G. P. , … Anderson, T. J . (2010). The MoCA: Well‐suited screen for cognitive impairment in Parkinson disease. Neurology, 75, 1717–1725. https://doi.org/10.1212/WNL.0b013e3181fc29c9 21060094 10.1212/WNL.0b013e3181fc29c9

[brb3792-bib-0005] Dauer, W. , & Przedborski, S. (2003). Parkinson's disease: mechanisms and models. Neuron, 39, 889–909. 12971891 10.1016/s0896-6273(03)00568-3

[brb3792-bib-0006] de Lau, L. M. L. , & Breteler, M. M. B. (2006). Epidemiology of Parkinson's disease. The Lancet Neurology, 5, 525–535. https://doi.org/10.1016/s1474-4422(06)70471-9 16713924 10.1016/S1474-4422(06)70471-9

[brb3792-bib-0007] Federico, F. , Simone, I. L. , Lucivero, V. , Iliceto, G. , De Mari, M. , Giannini, P. , … Lamberti, P . (1997). Proton magnetic resonance spectroscopy in Parkinson's disease and atypical parkinsonian disorders. Movement Disorders, 12, 903–909. 9399213 10.1002/mds.870120611

[brb3792-bib-0008] Gill, D. J. , Freshman, A. , Blender, J. A. , & Ravina, B. (2008). The Montreal cognitive assessment as a screening tool for cognitive impairment in Parkinson's disease. Movement Disorders, 23, 1043–1046. 18381646 10.1002/mds.22017

[brb3792-bib-0009] Goetz, C. G. , Tilley, B. C. , Shaftman, S. R. , Stebbins, G. T. , Fahn, S. , Martinez‐Martin, P. , … Movement Disorder Society UPDRS Revision Task Force . (2008). Movement Disorder Society‐sponsored revision of the Unified Parkinson's Disease Rating Scale (MDS‐UPDRS): Scale presentation and clinimetric testing results. Movement Disorders: Official Journal of the Movement Disorder Society, 23, 2129–2170. https://doi.org/10.1002/mds.22340 19025984 10.1002/mds.22340

[brb3792-bib-0010] Holshouser, B. A. , Komu, M. , Moller, H. E. , Zijlmans, J. , Kolem, H. , Hinshaw, D. B. Jr. , … Tosk, J. M . (1995). Localized proton NMR spectroscopy in the striatum of patients with idiopathic Parkinson's disease: a multicenter pilot study. Magnetic Resonance in Medicine, 33, 589–594. 7596261 10.1002/mrm.1910330502

[brb3792-bib-0011] Hu, M. T. , Taylor‐Robinson, S. D. , Chaudhuri, K. R. , Bell, J. D. , Morris, R. G. , Clough, C. , … Turjanski, N . (1999). Evidence for cortical dysfunction in clinically non‐demented patients with Parkinson's disease: A proton MR spectroscopy study. Journal of Neurology, Neurosurgery and Psychiatry, 67, 20–26. 10.1136/jnnp.67.1.20 PMC1736418 10369817

[brb3792-bib-0012] Levin, B. E. , Katzen, H. L. , Maudsley, A. , Post, J. , Myerson, C. , Govind, V. , … Mittel, A . (2014). Whole‐Brain Proton MR Spectroscopic Imaging in Parkinson's Disease. Journal of Neuroimaging, 24, 39–44. 23228009 10.1111/j.1552-6569.2012.00733.x PMC4593470

[brb3792-bib-0013] Lucetti, C. , Del Dotto, P. , Gambaccini, G. , Bernardini, S. , Bianchi, M. , Tosetti, M. , & Bonuccelli, U. (2001). Proton magnetic resonance spectroscopy (1H‐MRS) of motor cortex and basal ganglia in de novo Parkinson's disease patients. Neurological Sciences, 22, 69–70. 11487206 10.1007/s100720170051

[brb3792-bib-0014] Mazuel, L. , Chassain, C. , Jean, B. , Pereira, B. , Cladière, A. , Speziale, C. , & Durif, F . (2015). Proton MR Spectroscopy for Diagnosis and Evaluation of Treatment Efficacy in Parkinson Disease. Radiology, 278, 142764 10.1148/radiol.2015142764 26237591

[brb3792-bib-0015] Moffett, J. R. , Namboodiri, M. A. , Cangro, C. B. , & Neale, J. H. (1991). Immunohistochemical localization of N‐acetylaspartate in rat brain. NeuroReport, 2, 131–134. 1768855 10.1097/00001756-199103000-00005

[brb3792-bib-0016] Nie, K. , Zhang, Y. , Huang, B. , Wang, L. , Zhao, J. , Huang, Z ., … Wang, L . (2013). Marked N‐acetylaspartate and choline metabolite changes in Parkinson's disease patients with mild cognitive impairment. Parkinsonism & Related Disorders, 19, 329–334. 23238068 10.1016/j.parkreldis.2012.11.012

[brb3792-bib-0017] Sanes, J. N. , Dimitrov, B. , & Hallett, M. (1990). Motor learning in patients with cerebellar dysfunction. Brain: A Journal of Neurology, 113, 103–120. 2302528 10.1093/brain/113.1.103

[brb3792-bib-0018] Sharma, S. , Moon, C. S. , Khogali, A. , Haidous, A. , Chabenne, A. , Ojo, C. , … Ebadi, M . (2013). Biomarkers in Parkinson's disease (recent update). Neurochemistry International, 63, 201–229. https://doi.org/10.1016/j.neuint.2013.06.005 23791710 10.1016/j.neuint.2013.06.005

[brb3792-bib-0019] Taylorrobinson, S. D. , Turjanski, N. , Bhattacharya, S. , Seery, J. P. , Sargentoni, J. , Brooks, D. J. , … Cox, I. J . (1999). A proton magnetic resonance spectroscopy study of the striatum and cerebral cortex in Parkinson's disease. Metabolic Brain Disease, 14, 45. 10348313 10.1023/a:1020609530444

[brb3792-bib-0020] Tedeschi, G. , Litvan, I. , Bonavita, S. , Bertolino, A. , Lundbom, N. , Patronas, N. J. , & Hallett, M. (1997). Proton magnetic resonance spectroscopic imaging in progressive supranuclear palsy, Parkinson's disease and corticobasal degeneration. Brain: A Journal of Neurology, 120(Pt 9), 1541–1552. 9313638 10.1093/brain/120.9.1541

[brb3792-bib-0021] Watanabe, H. , Fukatsu, H. , Katsuno, M. , Sugiura, M. , Hamada, K. , Okada, Y. , … Sobue, G . (2004). Multiple regional 1H‐MR spectroscopy in multiple system atrophy: NAA/Cr reduction in pontine base as a valuable diagnostic marker. Journal of Neurology, Neurosurgery and Psychiatry, 75, 103–109. PMC1757481 14707317

[brb3792-bib-0022] Weingarten, C. P. , Sundman, M. H. , Hickey, P. , & Chen, N. K. (2015). Neuroimaging of Parkinson's disease: Expanding views. Neuroscience and Biobehavioral Reviews, 59, 16–52. https://doi.org/10.1016/j.neubiorev.2015.09.007 26409344 10.1016/j.neubiorev.2015.09.007 PMC4763948

[brb3792-bib-0023] Wu, G. , Shen, Y. J. , Huang, M. H. , Xing, Z. , Liu, Y. , & Chen, J. (2016). Proton MR Spectroscopy for Monitoring Pathologic Changes in the Substantia Nigra and Globus Pallidus in Parkinson Disease. AJR American Journal of Roentgenology, 206, 385–389. https://doi.org/10.2214/AJR.14.14052 26797368 10.2214/AJR.14.14052

[brb3792-bib-0024] Zanigni, S. , Testa, C. , Calandra‐Buonaura, G. , Sambati, L. , Guarino, M. , Gabellini, A. , … Tonon, C . (2015). The contribution of cerebellar proton magnetic resonance spectroscopy in the differential diagnosis among parkinsonian syndromes. Parkinsonism & Related Disorders, 21, 929–937. https://doi.org/10.1016/j.parkreldis.2015.05.025 26077167 10.1016/j.parkreldis.2015.05.025

