# Supplementary material for: Detection and application of neurochemical profile by multiple regional 1H‐MRS in Parkinson's disease
Source: Brain Behav. 2017 Aug 13;7(9):e00792. doi: 10.1002/brb3.792 (PMC5607555; doi:10.1002/brb3.792)
Supplement: Supplementary file 1 [file BRB3-7-e00792-s001.doc]

**ONLINE SUPPLEMENT Supporting Information File S1.**

**Figure legends**

**
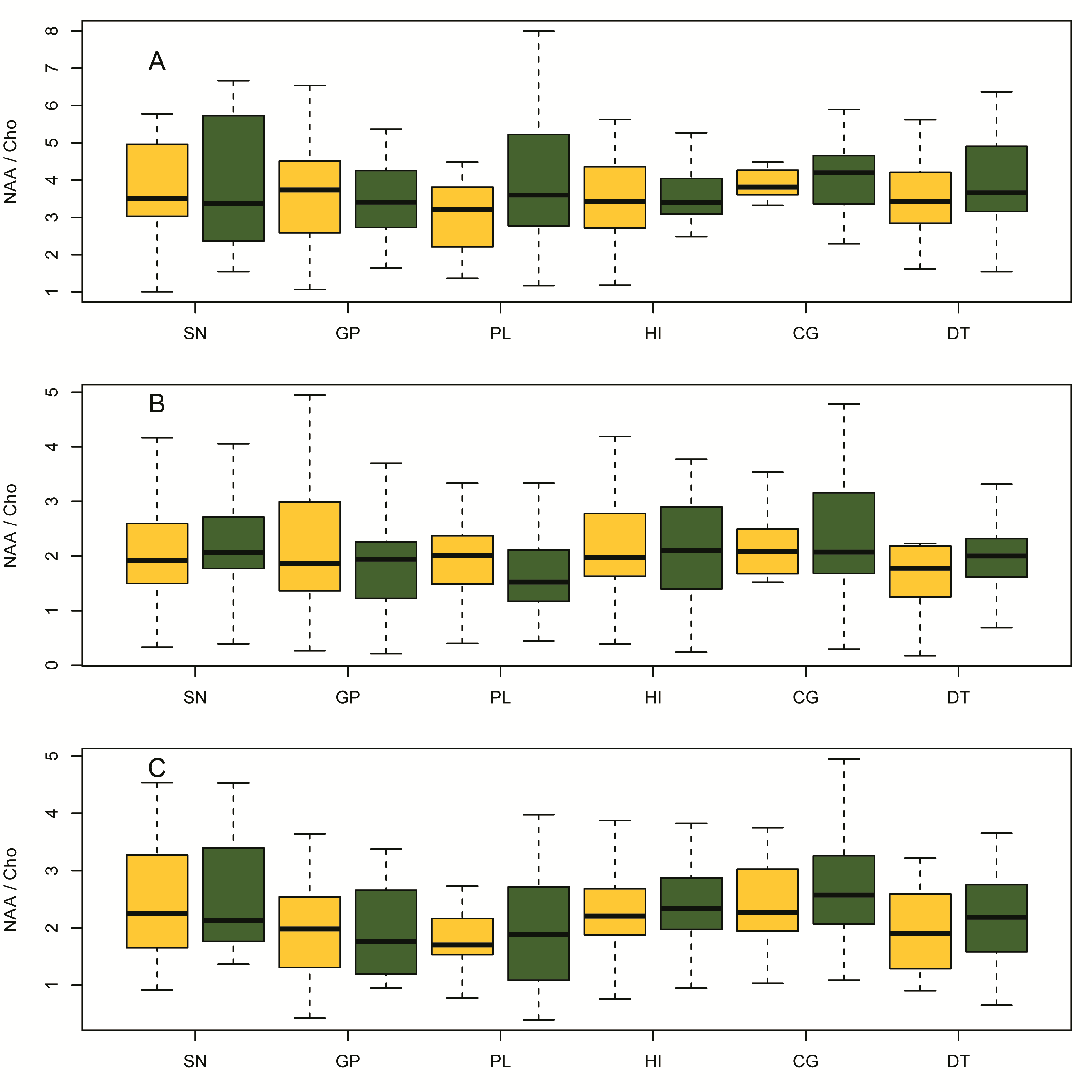
**

Fig A. NAA/Cho ratio in substantia nigra, globus pallidus, prefrontal lobe, hippocampus, cuneus gyrus and dorsal thalamus for A: healthy volunteers in left and right brain. B: PD patients with unilateral impairment in the brain contralateral or ipsilateral to clinical sign and C: PD patients with bilateral impairment in left and right brain.


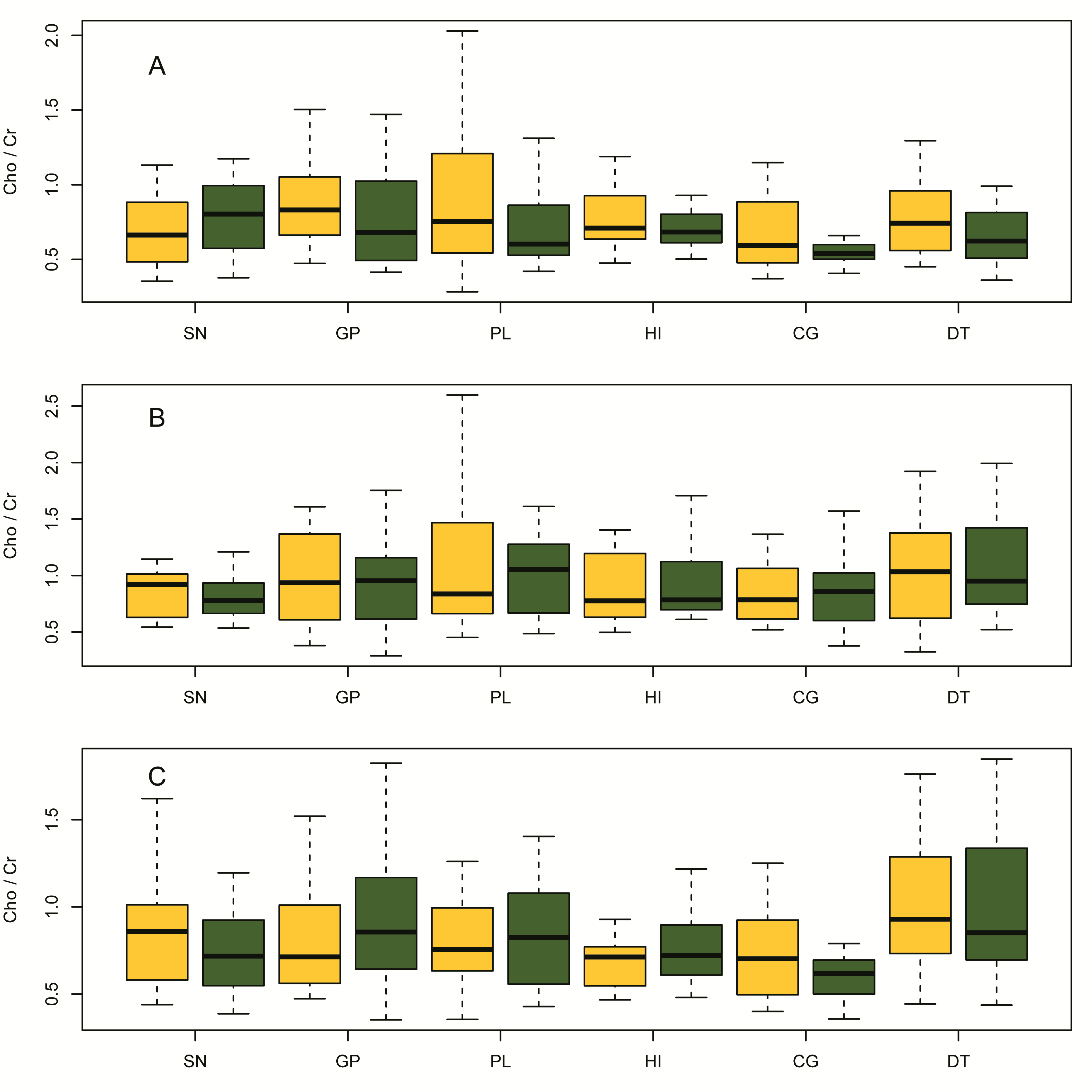


Fig B. Cho/Cr ratios in substantia nigra, globus pallidus, prefrontal lobe, hippocampus, cuneus gyrus and dorsal thalamus for A: healthy volunteers in left and right brain. B: PD patients with unilateral impairment in the brain contralateral or ipsilateral to clinical sign and C: PD patients with bilateral impairment in left and right brain.


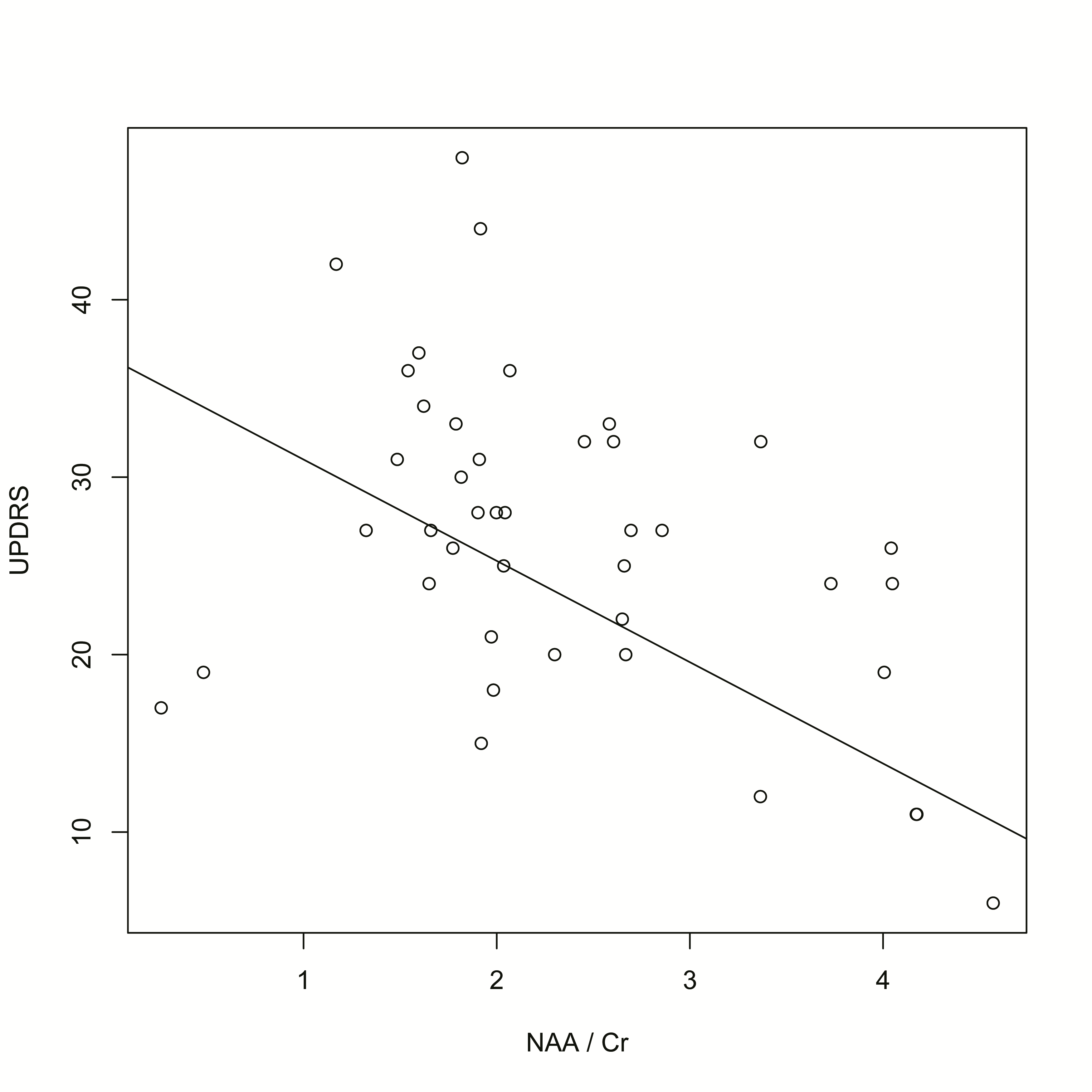


Fig C. Correlation between the Unified Parkinson’s Disease Rating Scale (UPDRS) and NAA/Cr ratio in the substantia nigra.


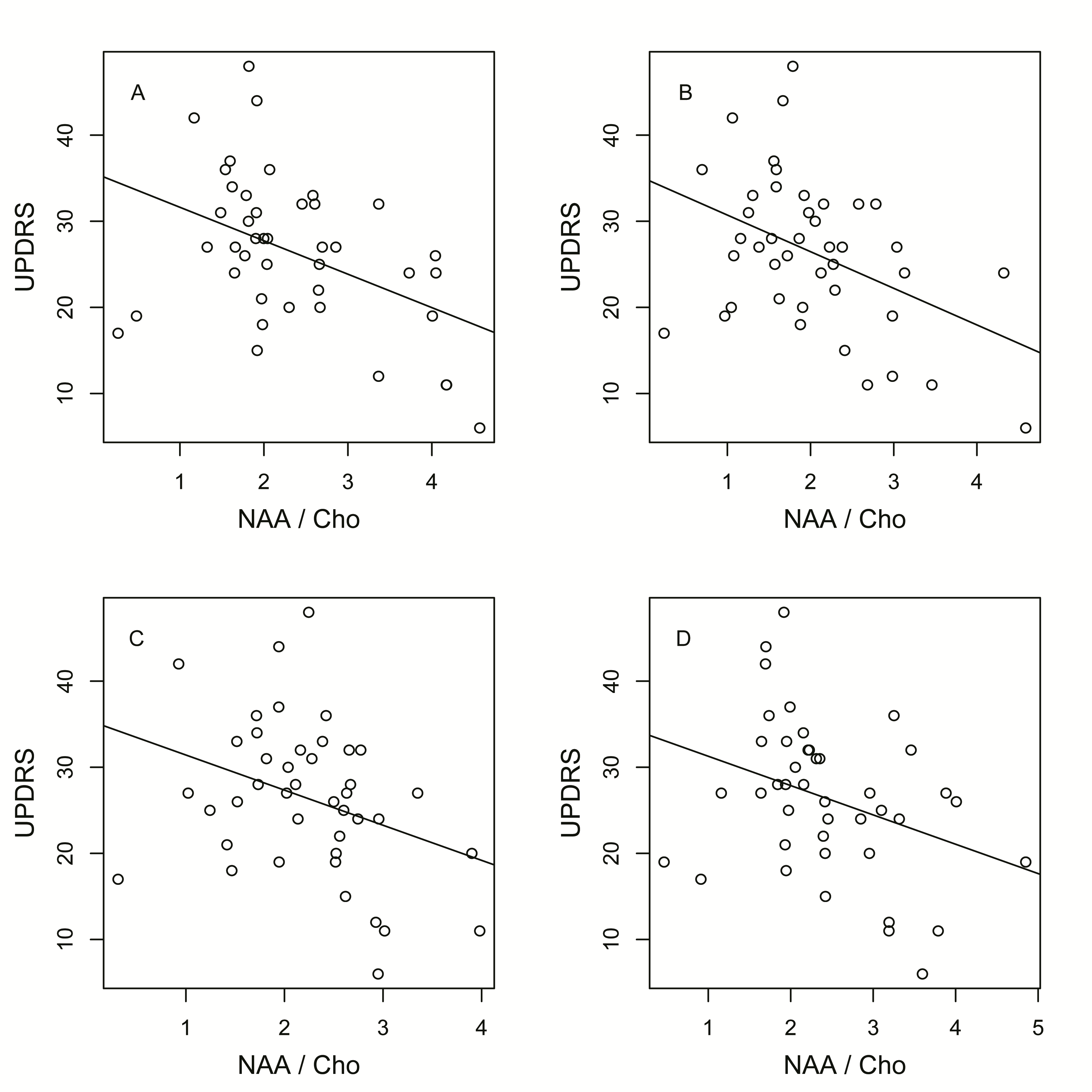


Fig D. Correlation between the Unified Parkinson’s Disease Rating Scale (UPDRS) and NAA/Cr ratios in substantia nigra (A), globus pallidus (B), hippocampus (C) and dorsal thalamus (D).
