# Supplementary figures and images for: Detection and application of neurochemical profile by multiple regional 1H‐MRS in Parkinson's disease
Source: Brain Behav. 2017 Aug 13;7(9):e00792. doi: 10.1002/brb3.792 (PMC5607555; doi:10.1002/brb3.792)

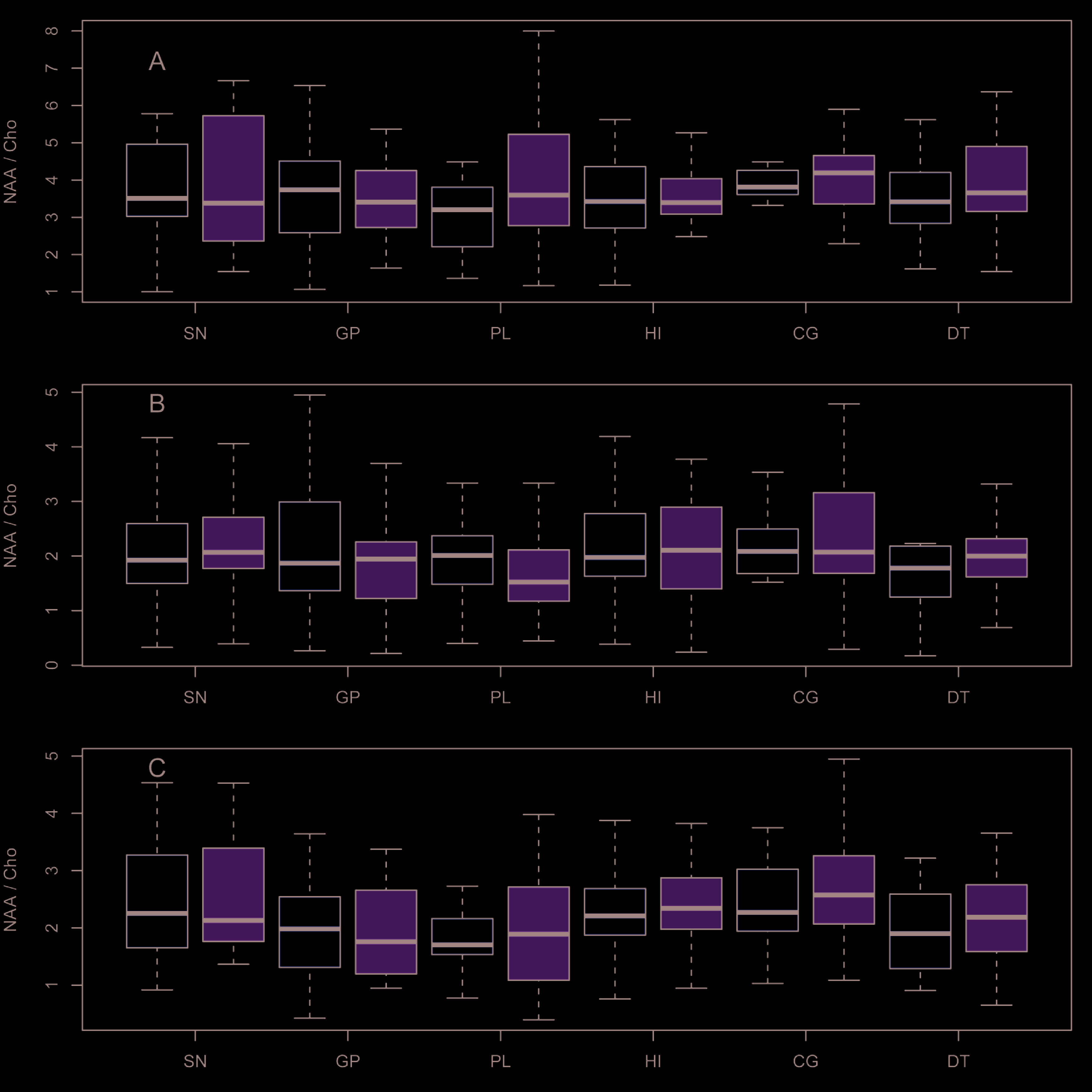

Supplement: Supplementary file 2 [file BRB3-7-e00792-s002.tif]

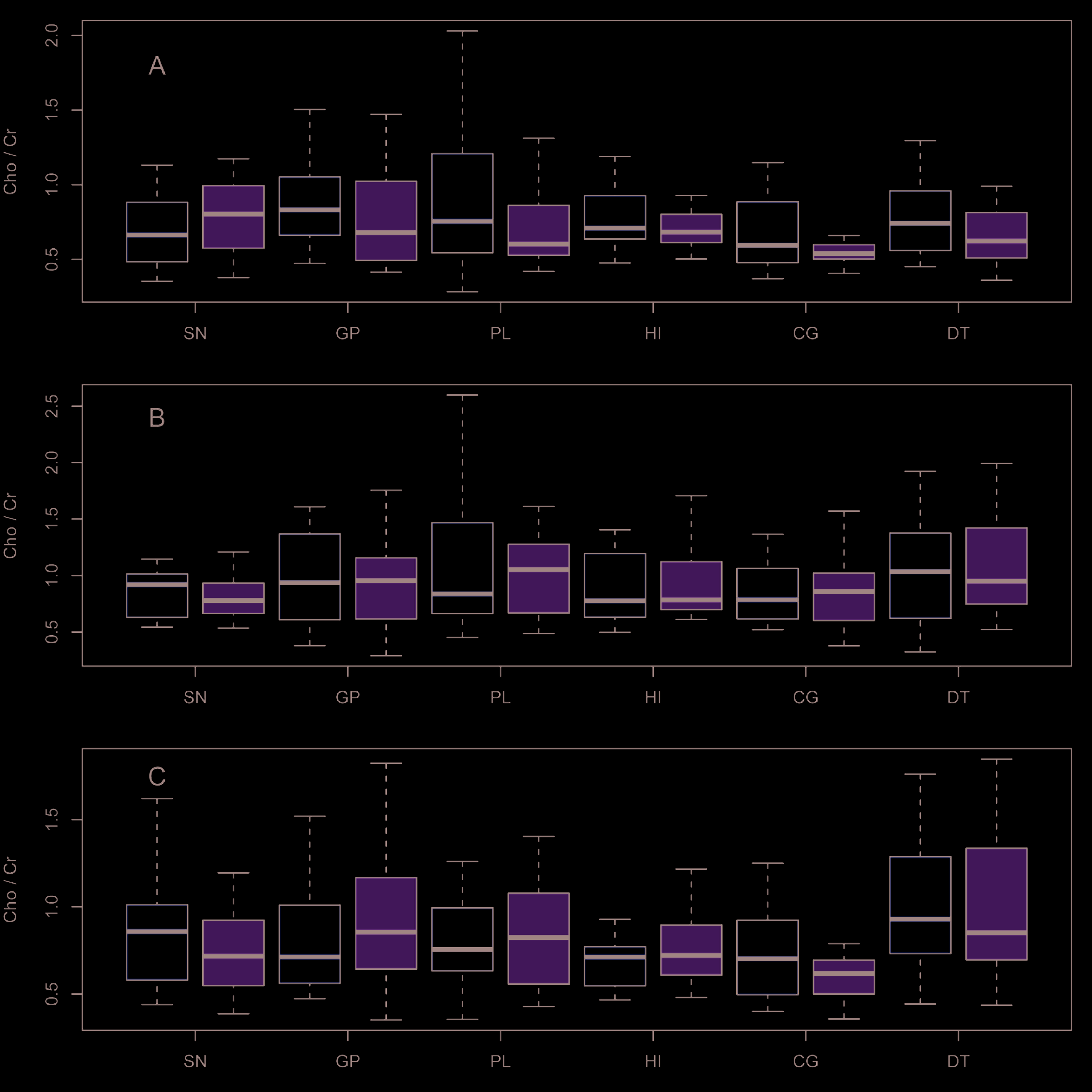

Supplement: Supplementary file 3 [file BRB3-7-e00792-s003.tif]

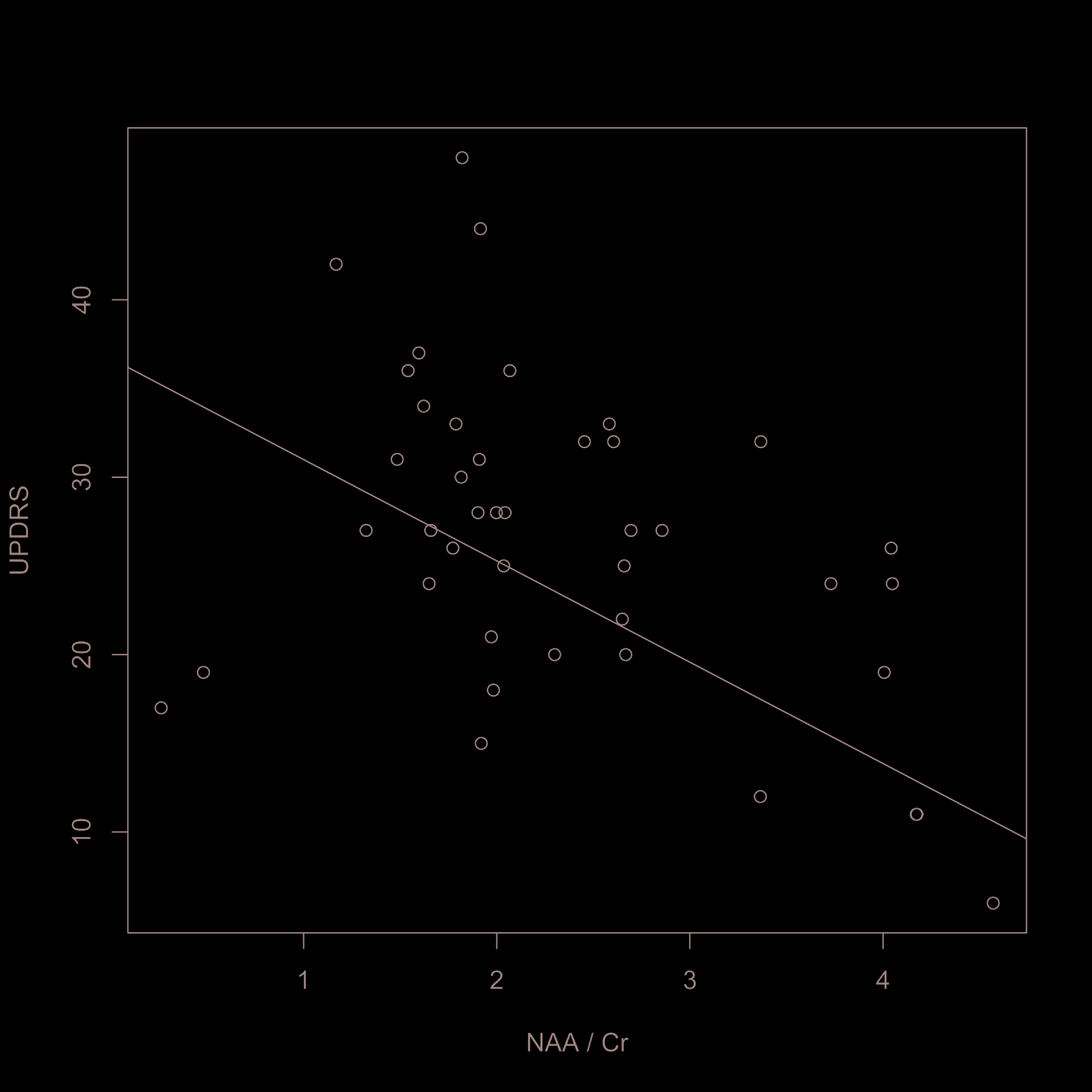

Supplement: Supplementary file 4 [file BRB3-7-e00792-s004.tif]

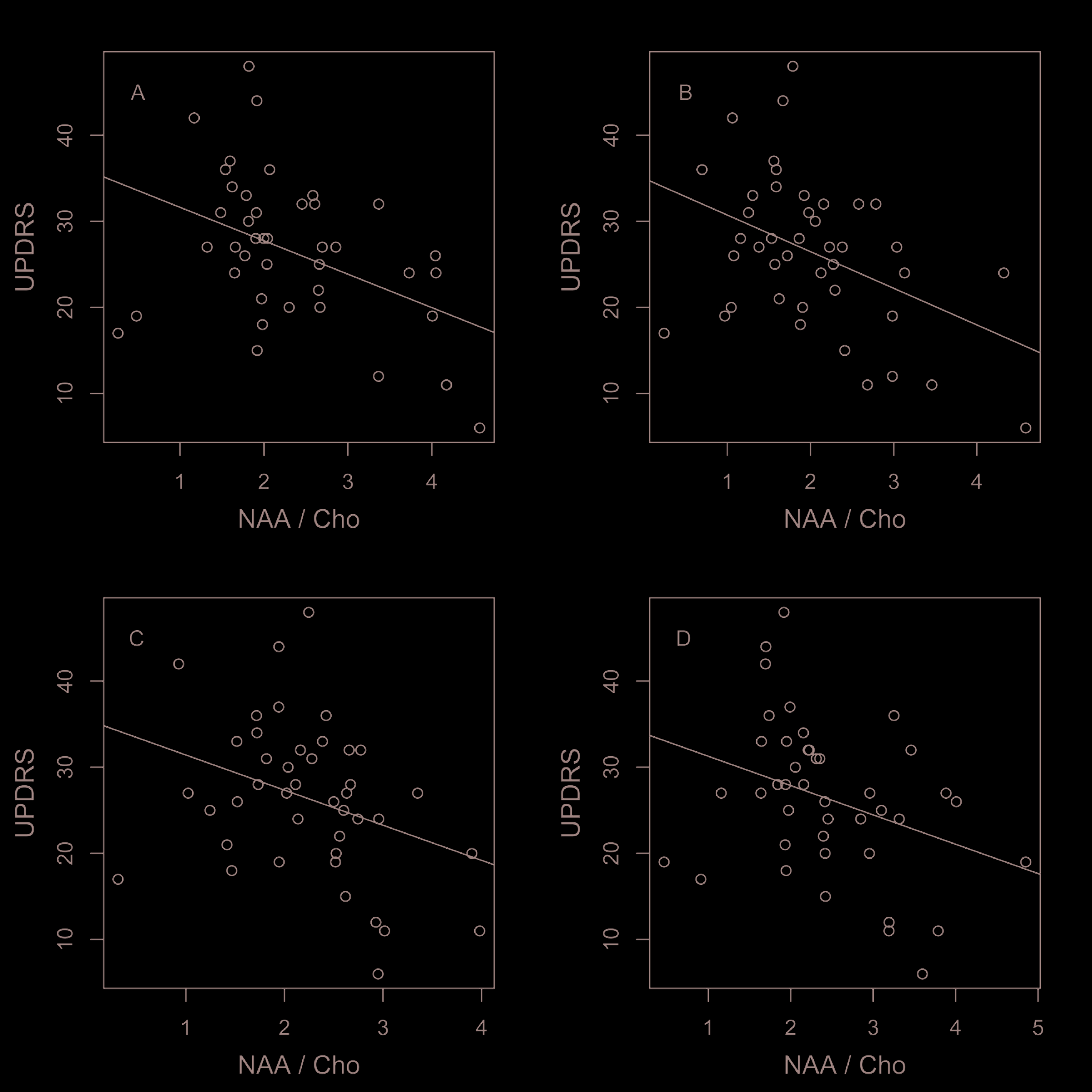

Supplement: Supplementary file 5 [file BRB3-7-e00792-s005.tif]
